# The Past, Present, and Promising Future of Direct Cardiac Compression Devices

**DOI:** 10.1016/j.jacbts.2025.02.013

**Published:** 2025-07-30

**Authors:** Melanie P. Hager, Paulamy Ganguly, George V. Letsou

**Affiliations:** a Texas A and M University, Department of Biomedical Engineering, College Station, Texas, USA; b Texas A and M University, School of Medicine, Bryan, Texas, USA; c Texas A and M University, School of Engineering Medicine, Houston, Texas, USA; d University of Houston College of Medicine, Houston, Texas, USA

**Keywords:** biventricular support, direct cardiac compression, heart failure, mechanical circulatory assist, ventricular assist devices

## Abstract

Direct cardiac compression (DCC) devices, under development as a new modality for mechanical cardiac support (MCS), offer several advantages over presently available forms of MCS. DCC devices avoid the blood contact obligatory with other implantable MCS devices, the complications associated with blood contact and hematologic incompatibility, such as thrombosis, stroke, and the need for anticoagulation are avoided, and DCC does not require vascular access eliminating challenges such as bleeding and extremity ischemia. Arterial pressure pulsatility is also maintained with DCC. Significant and underappreciated advancements in DCC technology have occurred over the last decades with notable dramatic improvements in cardiac performance and minimal tissue damage. One device has entered clinical trials with a second device anticipated to follow. DCC is poorly understood by most cardiologists and cardiac surgeons. This review summarizes DCC development and advances so that upcoming human clinical trials can be properly assessed.

## Highlights

• Newly developed DCC devices provide effective systolic and diastolic MCS; support can be for the LV, for the RV, or for both ventricles.
• Support of up to 1 month has been achieved.
• Neither thrombotic complications nor bleeding complications after DCC have been observed.
• DCC development is progressing rapidly and human clinical trials are beginning.
• It is important that HF clinicians are aware of and understand this potentially important technology.

Heart failure (HF) is the most common causes of death in developed countries with 500,000 to 1,000,000 new diagnoses of HF each year in the United States.^1^^,^^2^ HF therapies include medical regimens, surgical procedures such as coronary artery bypass surgery, coronary artery stenting, and valve surgery. Mechanical cardiac support (MCS) has been reserved for those most seriously affected.

MCS is currently limited by several factors. The relatively large size of implantable devices currently available make them difficult to place in smaller patients, limiting their use in women and children. Although women develop HF at the same rate as men, only 25% of implantable devices are placed in women.^3^ Current MCS devices are placed intravascularly, predisposing to thrombosis and requiring anticoagulation. Temporary devices such as the Impella and intra-aortic balloon pump require vascular access with attendant challenges of access vessel size and insertion site bleeding. Current MCS devices require urgent removal for device malfunction to prevent intravascular and systemic thrombotic complications. There is a pressing need for a less-invasive, non–blood contacting alternative to current MCS. Devices that are easier to insert, are smaller, do not require anticoagulation, and can be deactivated easily are required. Such devices would broaden MCS availability, especially to women and children who are currently underserved.

Direct cardiac compression (DCC) devices offer a novel means of MCS that address these issues. Significant advances in DCC technology have been made over the last 30 years but are not yet well appreciated by HF practitioners. A review of these advances is necessary to understand the impending human clinical trials.

## Early Circulatory Support and Cardiac Compression

Modern efforts at resuscitation and circulatory support began in the 1500s with the use of respiratory bellows after restrictions on resuscitation instituted during the Renaissance were discarded. The Scottish physician Tossach^4^ described mouth-to-mouth resuscitation in 1732. By the mid-1800s, German practitioners were investigating the role of cardiac compression, analogous to present day cardiopulmonary resuscitation.^5^

In 1877, Rudolph Boehm demonstrated that external cardiac compression restored circulation in cats.^6^ In 1903, George Crile, at the Cleveland Clinic, demonstrated that external cardiac compression restored canine circulation.^7^ Crile went on to document that closed-chest cardiac compression (CCC) was effective in humans, but the technique was not widely accepted.^7^

Turk and Glenn^8^ at Yale described 42 cases of human CCC in 1954. However, it was not until 1960 that further advances in ventilation and cardiac defibrillation allowed Kouwenhoven, Safar, and Jude to introduce what we know as cardiopulmonary resuscitation, combining mouth-to-mouth breathing with CCC.^9^

Although external CCC combined with mask ventilation became the treatment of choice for outpatient and inpatient resuscitation after cardiopulmonary arrest internal DCC has been significantly more difficult to accomplish.

## DCC From 1950 to Present

### 1950s and 1960s

**Figure 1:**
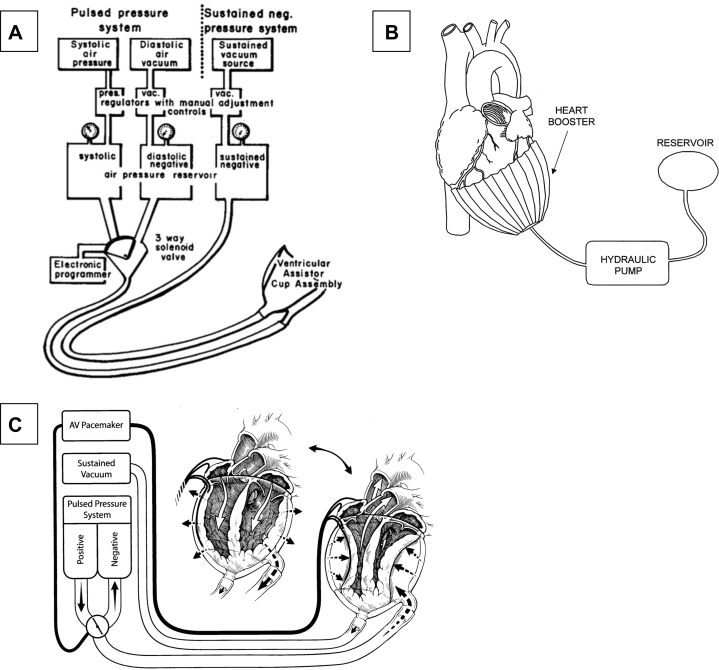
Schematics of Early DCC Devices (A) Schematic of the *Anstadt Cup*. Reprinted from Anstadt et al^11^ with permission from Elsevier. (B) Schematic of the *Heart Booster*. Reprinted from Kung et al^18^ with permission from Elsevier. (C) Schematic of the *updated Anstadt Cup* now featuring defibrillation electrodes. Reprinted from Anstadt et al^24^ with permission from Elsevier. AV = atrioventricular.

Bencini and Parola described one of the first implantable DCC devices in 1956, but their investigation was limited.^10^ Serious efforts at DCC device development began in the 1960s with what was termed “direct mechanical ventricular assist.” In 1966, Anstadt et al^11^ described direct mechanical ventricular assist using a glass assistor cup with a flexible diaphragm held onto the heart by suction. The glass cup was lined with a silicone diaphragm and featured an opening at the apex through which 60 mm Hg of suction was administered to keep the ventricles fixed in the cup. A device sidearm enabled administration of 140 mm Hg to −80 mm Hg of pressure in the cup to assist with systole and diastole, respectively (see Figure 1A). Throughout the 1960s, the group demonstrated preclinical efficacy of this device, dubbed the “Anstadt Cup,” in canines.^11^ By 1968, the Anstadt design had been used clinically for 1 hour to achieve resuscitation of a patient.^12^ This device was designed with resuscitation from full cardiopulmonary arrest as the goal; use as a cardiac assist device was not the objective.

### 1970s

Anstadt’s group continued device development in the 1970s. A platinum electrode was added to the device apex, allowing for defibrillation. This updated design successfully defibrillated canines without interruption of MCS (see Figure 1C). Prolonged MCS was provided for 3 days in an animal with ventricular fibrillation (VF).^13^

### 1980s

In the 1980s, Anstadt’s group continued to present promising data regarding DCC capabilities in resuscitation from cardiopulmonary arrest. The superiority of DCC relative to open-chest manual compression and CCC in canines during VF was documented. At a rate of 60 compressions per minute, DCC produced a mean arterial pressure (MAP) of 72 mm Hg during actuation, while open-chest manual compression and CCC produced MAPs of only 50 and 26 mm Hg, respectively.^14^ At this point, DCC was still envisioned primarily as a device for resuscitation from cardiopulmonary arrest.

### 1990s

In the 1990s, Anstadt et al^15^^,^^16^ continued to explore DCC for cardiopulmonary arrest. Studies in humans after cardiopulmonary arrest compared DCC to open-chest cardiac massage (OCCM); DCC increased systemic arterial pressure and cardiac output (CO) by 65% and 190%, respectively, compared with OCCM.^15^ In animal and human models, organ perfusion following DCC was improved compared with OCCM.^16^

The first DCC clinical trial using the Anstadt cup in 22 patients with refractory cardiopulmonary arrest was reported in 1991. The device restored pulsatile flow and generated a MAP of 78 ± 4 mm Hg. The device, which was inserted via thoracotomy, required <2 minutes from the time of skin incision to application and activation, was more effective than OCCM, and caused no cardiac damage.^15^

The Anstadt group demonstrated that DCC did not damage the heart, did not damage saphenous vein bypass grafts, and did not affect saphenous vein graft blood flow after coronary artery bypass graft. Using an acute canine model, saphenous vein graft bypasses were performed in 11 animals. During sustained induced VF, 5 animals were supported using DCC and 6 using CPB. DCC and CPB provided similar levels of hemodynamic support as assessed by the levels of inotropic support to maintain arterial pressure. Although electromagnetic flow probe graft flow analysis proved unreliable during DCC perfusion, microsphere injections did not demonstrate any significant myocardial perfusion differences. Histologic analysis demonstrated no damage to the saphenous vein grafts or anastomoses.^17^ Because coronary perfusion occurs primarily during diastole, these results are not unexpected, but they are reassuring.

Other groups began to develop DCC devices during the 1990s. Kung et al^18^ published a proof-of-concept paper describing the “Heart Booster” (AbioMed, Inc) in 1999 (see Figure 1B). The Heart Booster used tubular inflatable chambers to surround the left ventricle (LV) and right ventricle (RV) for LV support. Device effectiveness was demonstrated in 3 juvenile calves (70-80 kg). Implantation of the Heart Booster within the pericardial sac was achieved via median sternotomy and opening the pericardium. The device required suturing to the pericardium at the atrioventricular groove. Improvements in oxygen saturation and aortic pressure (AoP) were demonstrated with device actuation in normal calves. AoP increased by 15 mm Hg and CO increased by 10%.^18^ This further demonstrated DCC effectiveness and is one of the early attempts at using DCC for HF, rather than resuscitation from cardiopulmonary arrest.

**Figure 2:**
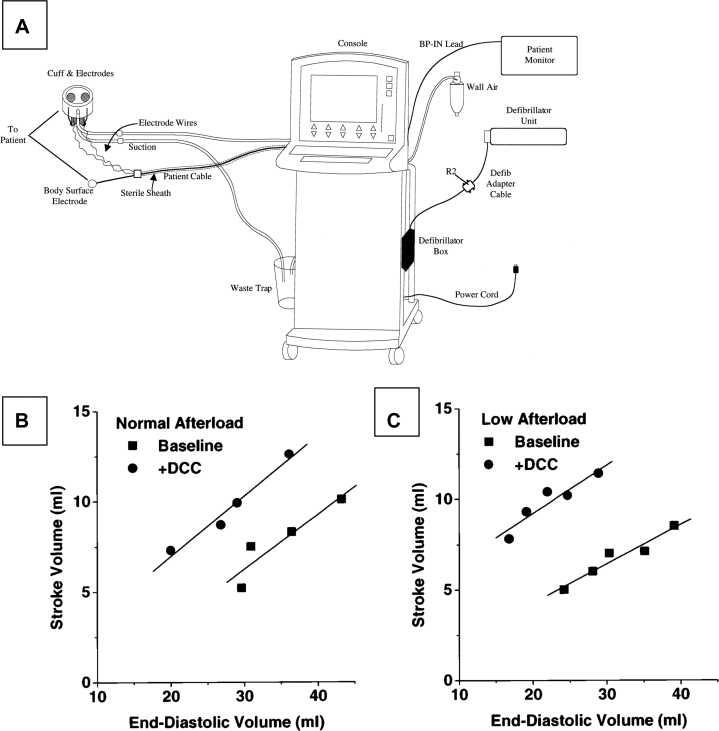
CardioSupport (A) Schematic of CardioSupport. Reprinted from Williams et al^22^ with permission from Elsevier. Effect of direct cardiac compression (DCC) on the relationship between end-diastolic volume and stroke volume at normal (B) and low (C) afterload arterial resistances from a representative heart. DCC shifts the end-diastolic volume–stroke volume relationship upward. Reprinted from Artrip et al^21^ with permission from Elsevier.

The CardioSupport System (Cardio Technologies Inc) was reported in 1999 and was intended for synchronized augmented compression. It consisted of a mechanical wrap of the heart that would contract in synchrony with the cardiac cycle. It was implanted via thoracotomy (see Figure 2) and adhered to the epicardial surface using approximately 200 mm Hg of suction at the cardiac apex. The inner surface included 2 electrodes for both synchronized pacing and cardioversion. The device had a rigid outer layer, a polyurethane inflation bladder, and a vacuum/inflation line. The CardioSupport device was among the first to activate in synchrony with the heart. Thus, its anticipated use was not only for resuscitation, but also for chronic cardiac assistance.^19^ Artrip et al,^20^ working with the CardioSupport System, demonstrated augmented end-systolic pressure without compromising myocardial oxygen consumption using DCC in an isolated canine HF model.

These studies in the 1990s established that DCC could provide highly effective MCS without cardiac damage in animals and that DCC could be effective for HF as well as for resuscitation.

### 2000s

In the 2000s, the CardioSupport System investigation continued. In an ex vivo canine model, the CardioSupport System DCC device improved CO to approximately 55% of normal. When filling pressures, preload, and afterload were manipulated in the ex vivo model, DCC restored CO to near normal. DCC resulted in increased the slope of the end-diastolic volume to stroke volume (SV) relationship at normal and low afterload conditions clearly documenting increased SV as seen in Figures 2B and 2C.^21^ The CardioSupport System was also assessed in vivo at inflation pressures of 50, 100, and 150 mm Hg in a canine acute HF model induced by microembolization. At 150 mm Hg, CO increased by 50% compared with the unassisted HF state, approximately 50% of the normal CO. This was the first demonstration of device inflation pressure’s importance in CO improvement. CO with DCC was also demonstrated to be dependent on afterload. As with the ex vivo heart studies, DCC effectiveness and the importance of adequate preloading was evident.^21^ In addition to the acute canine studies, the CardioSupport System was successfully chronically implanted in normal sheep without HF for up to 7 days.^22^

In the 2000s the Heart Booster was modified and renamed the “AbioBooster.” In 2001 sheep studies, the AbioBooster was implanted via an open left thoracotomy in 12 acute experiments and in 3 14-day survival experiments. The AbioBooster demonstrated effective MCS for up to 4 weeks in this configuration.^23^ Significant improvements in CO and LV SV occurred with DCC during the acute experiments. Significant improvements in CO and LV SV also were documented during episodically induced HF and induced VF at the conclusion of the 14-day survival experiments. Chronic HF was not investigated. Coronary artery bypass graft was performed in 6 of the 12 acute animals. Importantly, left internal mammary artery and saphenous vein graft flow were not affected by DCC in the 3 experiments where graft flow was assessed. The absence of any effect on graft flow is expected because, as noted previously, coronary artery flow occurs during diastole when DCC devices are decompressing. There were no detrimental effects noted at the surgical graft anastomoses at necropsy. The 3 DCC devices implanted for 14 days were all removed easily without evidence of adhesions to the underlying heart. There was no evidence of device adhesion to the epicardium at necropsy.

MCS for 3 hours duration in 8 cattle with beta-blocker–induced HF were assessed in 2002 by Anstadt et al.^24^ Although the trial’s DCC device did not activate synchronously with the cardiac cycle, appropriately timed DCC support was achieved by pacing the beta-blockaded heart at 100 beats/min and activating the DCC device at the same rate, timed to cardiac contraction. LV flow, RV flow, AoP, and pulmonary artery pressure (PAP) all returned toward baseline with DCC device activation. Only very limited device-induced tissue trauma was evident on histologic examination. Foci of myocyte necrosis and subepicardial hemorrhages were noted in the regions contacting the ports and cup rim, but the remainder of the myocardium did not show any evidence of damage.^24^

### 2010s

Building upon the concept of the AbioBooster device, Kavarana et al^25^ pursued development of the smaller PediBooster device for biventricular support in the setting of pediatric post-cardiotomy shock. The PediBooster achieved DCC using 2 balloons embedded in a mesh skeleton. However, preliminary swine studies demonstrated significant limitations, such as diastolic restriction and bruising of the myocardium. These difficulties were not noted with other devices and were likely related to the unique mesh skeleton of the PediBooster. Device design and avoidance of mesh is important in avoiding the detrimental structural effects seen with this device.

In a canine study with experimentally induced HF (n = 7), McConnell et al^26^ (in collaboration with Anstadt) found significant improvement in LV systolic and diastolic function following 2 hours of DCC support. The postsupport slopes of the preload-recruitable stroke work (+29% ± 7%; *P* < 0.05) and the end-diastolic pressure-volume relationship (−28% ± 9%; *P* < 0.05) reinforced the conclusion that short-term DCC improves cardiac contractility.^26^

### 2010 to Present

Experimental and clinical efforts clarified the hemodynamic effectiveness of several DCC device designs by 2010. The absence of thrombotic complications, lack of detrimental effects on cardiac structural integrity or saphenous bypass grafts, influence of inflation pressure on DCC effectiveness, and DCC’s potential had all been demonstrated. Efforts at development of an implantable device to treat HF rather than for resuscitation from cardiopulmonary arrest intensified. Several teams are now working towards this objective.

#### HeartPatch

**Figure 3:**
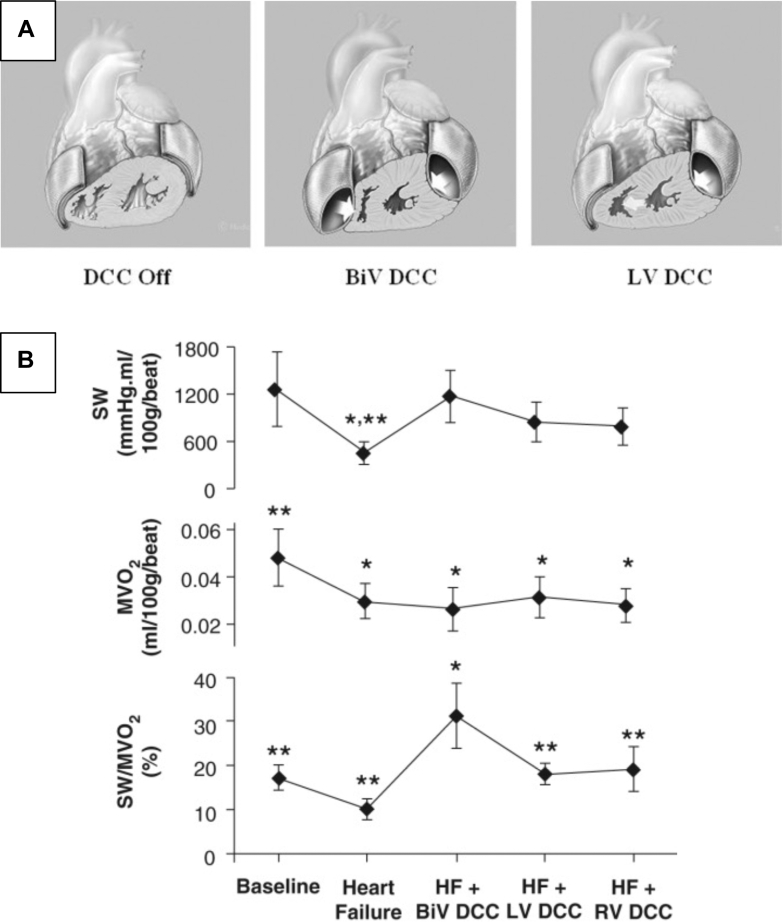
HeartPatch (A) Illustration of the direct cardiac compression (DCC) device on the heart demonstrating biventricular and left ventricle (LV) support. (B) Comparison of parameters at baseline, heart failure (HF), HF + DCC biventricular (BiV) support, LV support, and right ventricle (RV) support. Reprinted from Gallagher et al^28^ with permission from John Wiley and Sons. ∗*P* < 0.05 compared to baseline values; ∗∗ *P* < 0.05 compared to BiV DCC assist. n = 10 in each group. MVO2 = myocardial oxygen consumption; SW = stroke work.

The HeartPatch (Heart Assist Technologies Ltd) is an experimental DCC device with 2 silicone patches applied separately to the RV and LV epicardial surfaces (Figure 3A). Each patch has a visceral (epicardial)-facing and parietal (pericardial)-facing chamber. The parietal-facing chamber is sequentially inflated and deflated. The visceral-facing chamber provides negative pressure to enable adhesion of the chamber membrane to the epicardium. Mau et al^27^ evaluated the HeartPatch in 2 groups of 6 sheep: control and those with infarct-induced HF produced by septal coronary artery ablation. RV CO was measured in both groups, without DCC support, with passive placement, with single ventricle support, and with biventricular support. No significant changes in CO were observed. However, in the septal infarct group with DCC, higher CO was observed in all cases. RV end-diastolic dimension (EDD) to end-systolic dimension was reduced with LV DCC in the control group (*P <* 0.001) and with RV DCC in the septal infarct group (*P <* 0.001). RV stroke work also improved significantly with DCC. There was no evidence of diastolic restriction. The findings support the concept that DCC might also be important in recovery and remodeling after myocardial infarction.^27^

Gallagher et al^28^ studied the HeartPatch in 10 female sheep over at least 7 days treated with esmolol to induce HF. Hemodynamics were evaluated at least one week after initiation of DCC support. DCC utilizing the 2 HeartPatches on the LV and RV provided biventricular support, isolated LV support, and isolated RV support. LV end diastolic pressure (EDP) and RV EDP remained similar in the baseline and HF treatment groups except with isolated RV assist, which significantly elevated LV EDP (*P <* 0.05). LV efficiency (the ratio of stroke work to myocardial oxygen consumption) was significantly increased in the HF group with biventricular assist compared with the HF group with single ventricular assist (*P <* 0.05) (Figure 3B), further reinforcing the effectiveness of DCC for RV failure as well as LV failure.^28^

#### CorInnova

**Figure 4:**
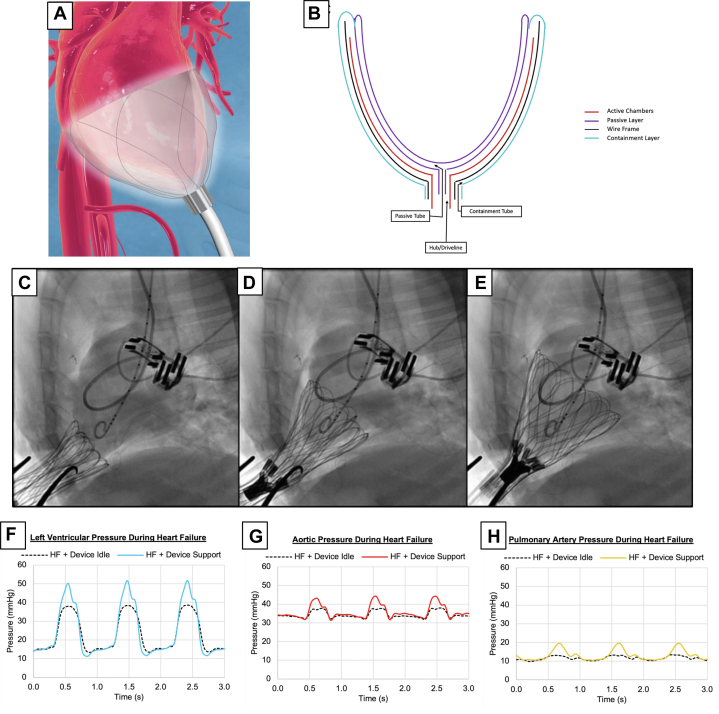
CorInnova (A) Illustration of device on cardiac model. (B) Illustration of device design demonstrating structure of layers. Reprinted from Letsou et al^29^ with permission from Elsevier. (C to E) Deployment of device illustrated with real-time imaging. Pressure tracings of left ventricle (F), aorta (G), and pulmonary artery (H) in heart failure (HF) state. Reprinted from Hord et al^32^ (CC BY 4.0, https://creativecommons.org/Licenses/by/4.0/).

The CorInnova device (CorInnova, Inc) is a minimally invasive DCC device that is deployed into the pericardial space without requiring sutures for fixation. The device has 2 layers of polyurethane chambers mounted on a self-expandable nitinol frame (Figures 4A and 4B). The inner chamber layer is filled with saline after deployment to intimately adhere to the ventricular surface. The air-filled outer chamber layer inflates and deflates in synchrony with the cardiac cycle using cardiac sensing electrodes mounted on the device.^29^ The device is inserted minimally invasively with fluoroscopic confirmation of correct device position (Figures 4C to 4E).

The CorInnova device is the first DCC device to demonstrate effectiveness in a chronic HF animal model. In a preclinical study using sheep with reduced ejection fraction (EF) (<30%), EF improved from 20% to 31% after 14 days of DCC in the experimental group (n = 3). When the device was turned off after 14 days in these 3 animals, the average EF remained significantly improved at 25%, indicating recovery of cardiac function after DCC. In the control group (n = 3), 2 animals expired before day 14; EF in the surviving animal was reduced to 17% on day 14.^30^

In a CorInnova preclinical acute ovine study, synchronous compression of the LV increased systolic pressure as documented by the left ventricular pressure (LVP) and AoP waveforms. These waveform changes were (as with an intra-aortic balloon pump) reliably present throughout the experiment’s course.^31^

In addition to promising data for adult applications, the CorInnova DCC device has demonstrated effectiveness in a pediatric proof-of-concept study using a miniaturized 9-chamber version of the CorInnova DCC device in an esmolol-induced acute HF goat model. In this study, the device recovered 95% of the SV compared with pre-HF baseline SV. Representative waveforms of increases in LVP, AoP, and PAP are shown in Figures 4F to 4H. Additionally, the pediatric-sized device did not demonstrate diastolic restriction^32^; diastolic restriction was a notable limitation of prior pediatric-sized DCC attempts.^25^^,^^33^

Preclinical CorInnova DCC animal trials have not revealed any cardiac damage, thrombotic episodes, bleeding, or arrhythmias. The device is being prepared for adult human HF clinical trials.^29^

#### AdjuCor GmbH

**Figure 5:**
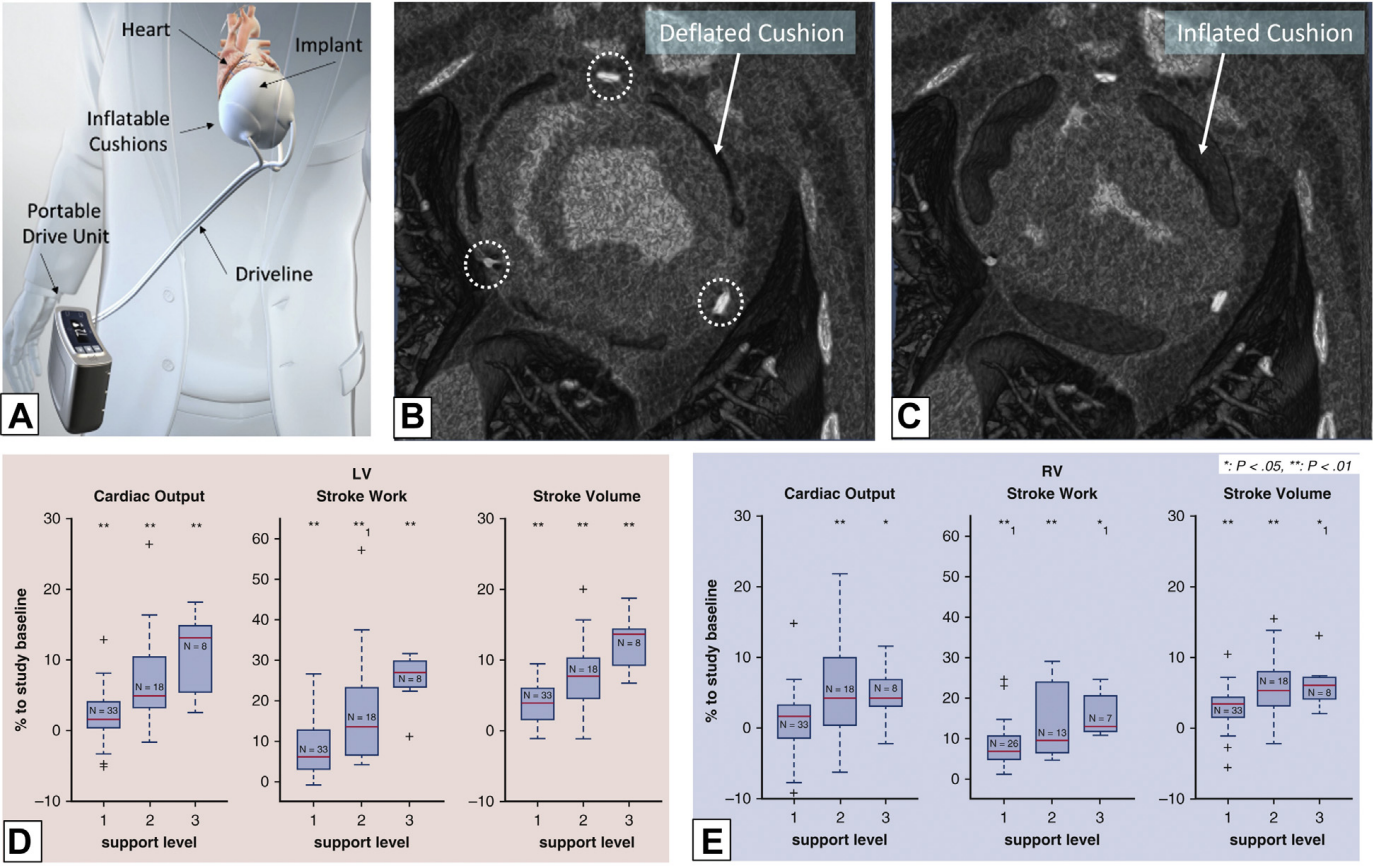
AdjuCor reBEAT (A) Illustration of device setup. (B-C) Deflated and inflated device cushions visualized in an axial view CT scan of the heart 16 days after implant. Dotted circles indicate the epicardial electrodes. (D) Increases in left ventricular (LV) cardiac output, stroke work, and stroke volume in an acute heart failure model as well as (E) effects on right-sided parameters. Reprinted from Schueler et al^34^ with permission from Elsevier. RV = right ventricle.

The reBEAT is an intrapericardial DCC device manufactured by AdjuCor GmbH. The device has 3 inflatable bladders: 2 interfacing with the LV and 1 interfacing with the RV (Figure 5A). The device offers 3 actuation modes: biventricular, LV, and RV assist. Bladder inflation and deflation is synchronized with electrocardiogram R and T waves, respectively. In animals, the device has been placed via a subxiphoid incision with wide opening of the pericardium to enable placement of the manually compressed device.^34^

A 2022 study assessed biventricular support in an HF model and 30-day device safety in animals without HF. Acute device safety and efficacy was demonstrated using 5 pigs (81-96 kg) and a beta-blocker–induced HF model. Actuation of the device during HF significantly increased CO and significantly decreased EDP (see Figure 5B). Device safety was further assessed in pigs without HF. The device remained implanted and activated for 30 days. After 30 days, postmortem macroscopic analysis of the myocardium showed tissue-device integration; no inflammation or infection was noted.^34^ Initial human clinical implantation of the device has been reported to be successful,^35^ but data on efficacy has not yet been presented.

#### Roche et al

**Figure 6:**
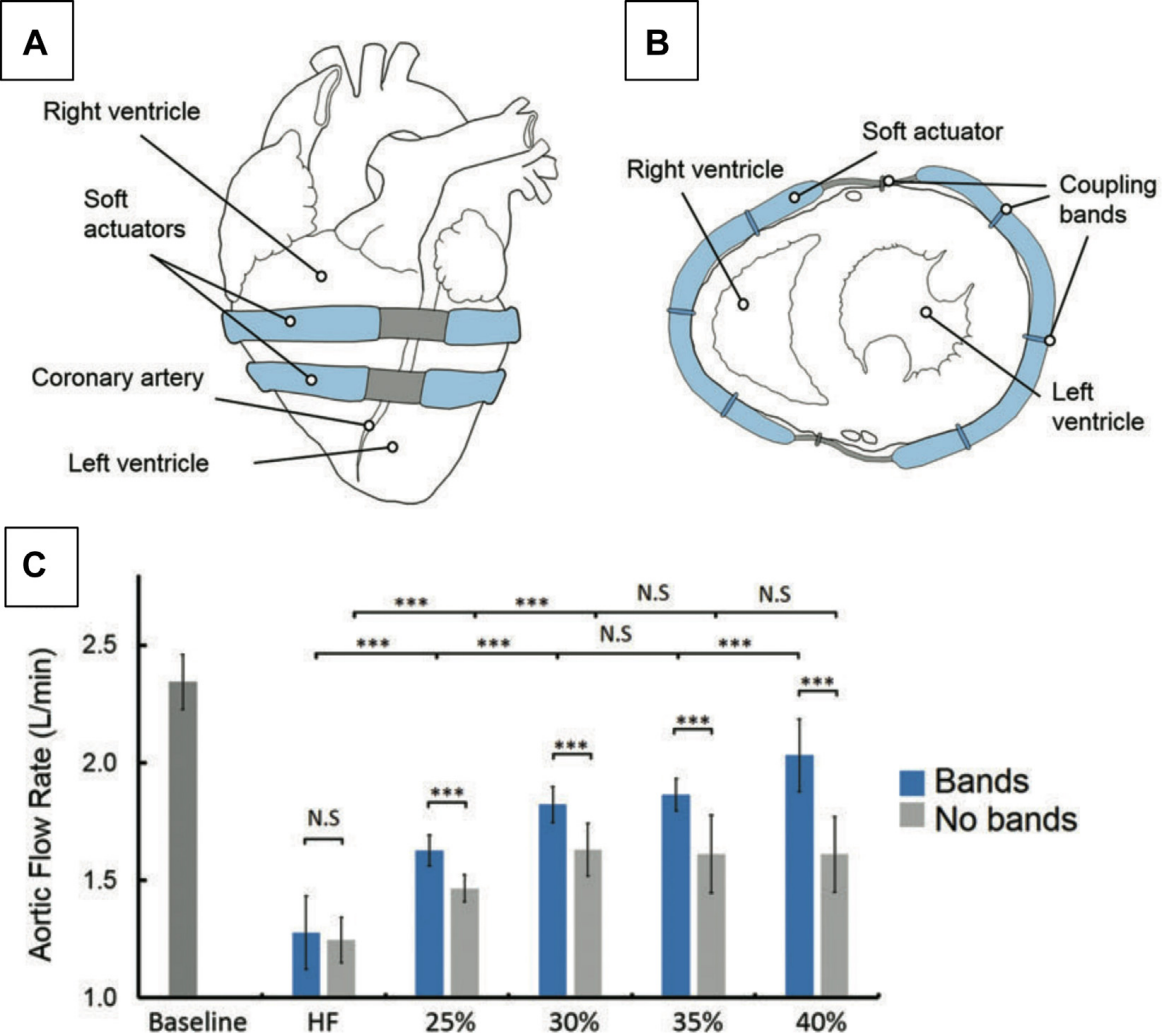
Direct Cardiac Compression Device (A and B) Schematic of device with inflatable bands in frontal and axial view. (C) Aortic flow rate compared in baseline, heart failure (HF), and variable device support. ∗∗∗*P* < 0.05. Reprinted from Payne et al^38^ with permission from Mary Ann Liebert, Inc.

Significant DCC progress is also being made by Roche’s group^36^^,^^37^ using advanced fixation techniques and sleeve designs (see Figure 6A). Roche’s design uses pneumatically actuated bladders embedded in silicone which are wrapped around the heart. External soft actuators are combined with septal bracing to engage the septum, maximizing LV and RV assistance. Elastic elements within the silicone function to provide active assistance of diastolic relaxation.^38^ The inflatable bladders are held in place with polyurethane mesh, a silicone passive restraint sleeve, and tissue-integration coupling the sleeve to the epicardium with either cyano-acrylate adhesive dye or Velcro; tissue integration and mechanical characterization of different fixation methods was performed in murine models. Swine with esmolol-induced HF had CO restored to normal in acute experiments using the device.^36^ Human clinical use has not been reported.

## Related Cardiac Compression and Cardiac Restraint Technologies

A complete discussion of technologies related to direct cardiac compression is not possible in this limited space. However, a brief discussion of dynamic cardiomyoplasty, of the CorCap cardiac restraint device, and of the PercAssist device are in order because of their close relation to mechanical DCC.

### Dynamic Cardiomyoplasty

Dynamic cardiomyopathy is a technique that involves wrapping the latissimus dorsi about the heart to provide synchronous direct cardiac compression. Electrical stimulation of the latissimus dorsi with proper synchronization led to systolic pressure generation of as much as 80 mm Hg during induced VF in an animal model. Human dynamic cardiomyoplasty did not produce demonstrable alterations in the arterial pressure waveform, possibly because of difficulties synchronizing skeletal muscle contraction to the cardiac cycle because of delays in skeletal muscle nerve conduction, but significant modest left ventricular ejection fraction (LVEF) improvements occurred.^39^^,^^40^ Notably, significant improvements in NYHA functional class were documented despite only modest improvements in LVEF. One possible explanation for the improvement in NYHA functional class was the decrease noted in LV volume after cardiomyoplasty. This led to the hypothesis that the most important effect of dynamic cardiomyoplasty might be a “girdling” effect preventing progressive LV dilation.^41^ A relatively high 30-day mortality of 3% to 10% in the absence of any documented improvement in survival led to the abandonment of clinical efforts with the procedure. However, the positive cardiomyoplasty experimental and clinical trial results influenced and reinforced findings from DCC investigations.

### Acorn CorCap

The possible importance of preventing LV dilatation postulated after the dynamic cardiomyoplasty experience was partially responsible for the development of a mesh polyester jacket that was placed around the ventricle. The Acorn CorCap (Acorn Cardiovascular, Inc) was designed to prevent and possibly decrease LV EDD in HF patients. Initial human clinical experience showed that LV EDD decreased as early as 3 months after implantation and was associated with improvements in LVEF. Significant improvements were maintained at 2 years.^42^

A substantial clinical study of 48 patients with dilated cardiomyopathy documented a 4.6% mean intraoperative reduction in LV EDD, LVEF improvement, and improvement in NYHA functional class without device-related intraoperative complications or evidence of long-term restrictive cardiac physiology.^42^ The results of a 5 year randomized trial documented significant reductions in LV EDD and improvements in NYHA functional class, but there was no survival benefit.^43^ The CorCap mesh device resulted in substantial scarring and pericardial/cardiac adhesions. Despite the beneficial effects on LV EDD and LVEF, U.S. Food and Drug Administration approval was withheld because of concerns over the possibility of severe pericardial/cardiac adhesions, the possibility of constrictive cardiac physiology, the lack of a survival advantage in the randomized trial, and the lack of statistically significant NYHA functional class improvements.

### PercAssist

PercAssist Inc began work on a minimally invasive, non–blood-contacting MCS in 2019. The PercAssist device improves CO by inflation and deflation of a balloon catheter within the pericardial sac. The balloon is advanced into the pericardial sac, anchored to the pericardium, and then inflated in synchrony with the patient’s ECG. First-in-human clinical studies (n = 2) were reported in early 2024.^44^ Initial EF was improved from 30% to 45% in patient 1 and 5% to 30% in patient 2. The balloon was implanted for 5 days and no adverse events were reported.^45^

## Comment

The “Anstadt cup” was the first extensively investigated implantable DCC device. It proved that effective cardiac compression could be provided by DCC when applied to a fibrillating heart. There was no cardiac damage after DCC, which might have been expected given the absence of cardiac damage seen after open-chest manual compression in most cases. The absence of any effects on saphenous vein bypass blood flow after coronary artery bypass were also experimentally documented. Several groups in the 1990s demonstrated improved cardiac performance in animal HF models using devices that required suturing to the heart to maintain proper device orientation. All these efforts led to the recognition that effective DCC was possible, but still problematic as the devices had a tendency to migrate or slip and alterations in the arterial pressure waveforms were not reliably documented.

Over the past 20 years, simpler methods for securely positioning DCC devices have been developed, the importance of synchronous device actuation with the cardiac cycle has been recognized, minimally invasive techniques for device insertion have been developed, and the CorInnova device has produced reliable reproducible alterations in systemic arterial and pulmonary arterial waveforms. It has also been documented that DCC devices are capable of RV as well as LV circulatory support. Survival studies in animals with experimentally induced chronic HF have documented improvements in LVEF and possibly, survival. Initial clinical trials of the AdjuCor device have started. The CorInnova and AdjuCor devices are illustrated in the Central Illustration along with the typical hemodynamic improvements of the CorInnova device in heart failure animals.

**Central Illustration:**
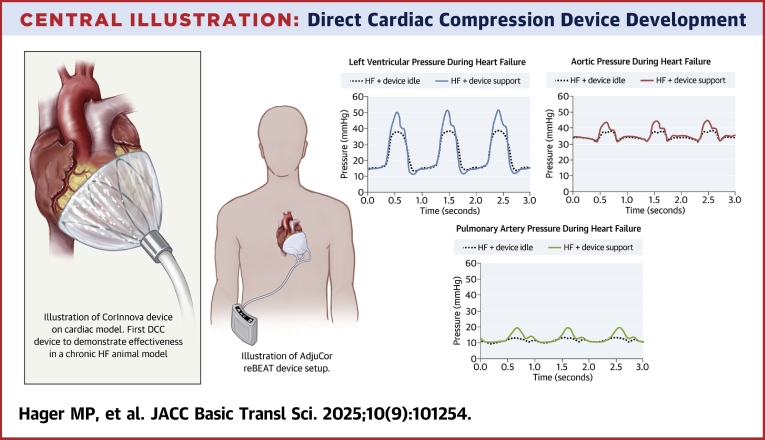
Direct Cardiac Compression Device Development Novel implantable direct cardiac compression devices include the CorInnova and AdjuCor reBeat devices that are entering human clinical trials. Hemodynamic effects of the CorInnova device in a HF animal model are illustrated on the right.

Controversial areas in DCC include whether the devices provide effective cardiac compression, whether the devices cause cardiac injury, whether the devices cause pericardial adhesions as well as the extent of adhesions, and whether the devices might migrate. To date, significant cardiac injury has not been documented in any of the extensive trials. None of the experimental trials have documented cardiac injury or direct injuries to the myocardium. In fact, one remarkable aspect of the animal studies to date is the lack of injury. Animal experiments have extended up to 60 days without documentation of any detrimental effects on cardiac integrity; effects beyond 60 days remain to be determined.

The lack of detrimental effects on cardiac function and structural integrity is not surprising. DCC devices contract in synchrony with the heart. When the heart is contracting, a DCC device is inflating, compressing the heart. When the heart is relaxing, a DCC device is deflating. DCC devices do not “fight” against the regular cardiac contractions, minimizing the risk of any direct myocardial damage. Another concern is DCC’s potential to inhibit coronary artery flow or damage surgically constructed bypass grafts. Coronary artery flow occurs during diastole, when DCC devices are deflating; therefore, compromised coronary artery flow in the native coronary arteries would not be expected. The lack of any documented coronary artery flow compromise or damage is consistent with cardiac physiology.

Concerns about pericardial adhesions arose from experience with the CorCap device. This device, manufactured using a polyester mesh, was purposely designed to adhere intimately to the epicardial surface to prevent ventricular distension. The device required snug wrapping around the ventricle and suturing to the epicardium to maintain proper positioning. Although published clinical studies did not reveal any increased mortality or device related complications in patients undergoing CorCap placement, the U.S. Food and Drug Administration did not approve the device at least partially based upon a review of extensive pericardial adhesions after device placement. Extensive pericardial adhesions were not noted in the human clinical trial of dynamic cardiomyoplasty. Cardiac adhesions were noted in patients with dynamic cardiomyoplasty for as long as 3 years, but the adhesions were not extensive, nor did they preclude further cardiac surgery or transplantation. In extensive experimental experience with DCC in animals, problematic pericardial adhesions have not been encountered. Concerns about pericardial adhesions after DCC for <30 days appear to be overstated.

Documentation of clearly beneficial hemodynamic effects on the arterial pressure waveform have been lacking until recently. Nearly all previous trials showed improvements in cardiac performance as assessed by CO or LV performance, but documentation of arterial pressure waveform effects was absent. Early DCC devices did not contract synchronously with the heart. With the development of devices capable of synchronizing with the cardiac cycle, further improvements in DCC hemodynamic efficacy were possible. Recent results with the synchronously contracting CorInnova device show reliable reproducible effects. We have proposed the term “copulsation” to describe the effects of DCC (in distinction to the “counterpulsation” seen with intra-aortic balloon devices where device activation occurs while the heart is relaxing). The increased systemic pressure and pulmonary arterial pressure seen with the CorInnova device can be thought of as “systolic augmentation” and the decreased pressures seen with cardiac relaxation as “diastolic unloading.”

Device positioning and maintenance of proper position has been markedly advanced through recognition of the pericardium’s potential in maintaining the DCC device’s position. The CorInnova device, which can be inserted in a minimally invasive fashion, does not require anchoring sutures because it is held in place by its self-expanding nitinol frame and an intact pericardium. Device dislodgement has not been reported in trials of the 2 devices currently being prepared for clinical use, the AdjuCor and CorInnova devices.

Thus last 50 years have led to several conclusions concerning DCC:

1. Appropriately designed implantable DCC devices provide effective systolic and diastolic MCS.
2. Appropriately designed implantable DCC devices can provide biventricular MCS.
3. Cardiac damage from implantable devices is minimal; significant damage has not been observed in any study.
4. Thrombotic complications after DCC have not been observed.
5. Bleeding complications from DCC have not been observed.
6. Prolonged support of up to 1 month has been reproducibly achieved using several devices.
7. Inflation chamber pressure influences the amount of cardiac support.
8. Restrictive cardiac physiology (similar to pericardial tamponade) has not been observed in any trial.
9. Sutureless fixation of DCC devices is possible.
10. Inflation and deflation of DCC devices synchronously with the cardiac cycle improves MCS.
11. Insertion of the device via mini thoracotomy leaving the pericardium intact may be important in appropriate device alignment and hemodynamic support.
12. Reproducible hemodynamic effects on the LVP, AoP, and PAP waveforms have been achieved.

DCC has many potential advantages over other methods of MCS. The devices can be implanted minimally invasively and do not require suturing to maintain proper position. Current DCC devices that can be inserted via minimally invasive incisions are also amenable to percutaneous placement in the catheterization laboratory. DCC devices can be turned off without detrimental effects on the recipient because they are not in the bloodstream, allowing the subject to rest, ambulate, recuperate, or sleep comfortably.

DCC has potential utility for patients for whom other forms of MCS are not feasible. The devices will make MCS available to patients who may not be candidates for other forms of MCS because of size (such as women and pediatric patients), contraindications to anticoagulation necessary for present forms of MCS, or difficulties with vascular access necessary for present forms of MCS. The absence of a blood-device interface eliminates device thrombosis risk, the attendant need for chronic anticoagulation, and the risk of bleeding associated with chronic anticoagulation. The ability to implant DCC devices in smaller patients may contribute substantially to the availability of MCS in women with HF and to the availability of pediatric MCS. Vascular access challenges, common with presently available MCS techniques, are eliminated with DCC devices. DCC devices may be useful in resource-limited countries because of lower anticipated cost when compared with other forms of MCS.

In summary, DCC devices provide significant, reproducible, and safe augmentation of cardiac performance in animal HF models without blood contact and without anticoagulation. Recent DCC development is progressing rapidly. Important human clinical trials are beginning. There is much reason for optimism regarding current DCC clinical trials and others that will be commencing in the near future. Additional attention should be dedicated to addressing gaps in the current understanding of DCC. It is important that HF clinicians are aware of and understand this potentially important technology.

## Funding Support and Author Disclosures

Dr Letsou is a full-time employee of TransMedics, Inc, Andover, MA, USA; and is a member of the CorInnova Scientific Advisory Board. All other authors have reported that they have no relationships relevant to the contents of this paper to disclose.
